# Supplementary material for: TerC proteins function during protein secretion to metalate exoenzymes
Source: Nat Commun. 2023 Oct 4;14:6186. doi: 10.1038/s41467-023-41896-1 (PMC10550928; doi:10.1038/s41467-023-41896-1)
Supplement: Supplementary file 1 — Supplementary Information [file 41467_2023_41896_MOESM1_ESM.pdf]

Supplementary Information

TerC Proteins Function During Protein Secretion to Metalate Exoenzymes

Bixi He[1], Ankita J. Sachla[1], and John D. Helmann[1*]

[1]Department of Microbiology, Cornell University, 370 Wing Hall, 123 Wing Drive, Ithaca, New York 14853-8101, USA

Supplementary Figure 1. Comparative growth of WT and FY mutants.

Supplementary Figure 2. Extracellular protease activities in the supernatants of mutant strains.

Supplementary Figure 3. Metal ion levels (μM) measured in cell supernatant fractions.

Supplementary Figure 4. Effect of *meeF*, *meeY* and FY mutations on AprE and AmyQ secretion.

Supplementary Figure 5. Maximum P_{htrA}-lux promoter activity in stains with or without AmyQ overexpression

Supplementary Figure 6. MeeF-FLAG and MeeY-FLAG levels in different mutants.

Supplementary Figure 7. Comparative phenotypes of mutations affecting protein secretion.

Supplementary Figure 8. Relative transcription level of LTA synthase genes in different strain backgrounds.

Supplementary Figure 9. Effects of metal ions on sensitivity to the LtaS inhibitor 1771.

Supplementary Table 1. *Bacillus subtilis* stains, primers, and plasmids used in this study.

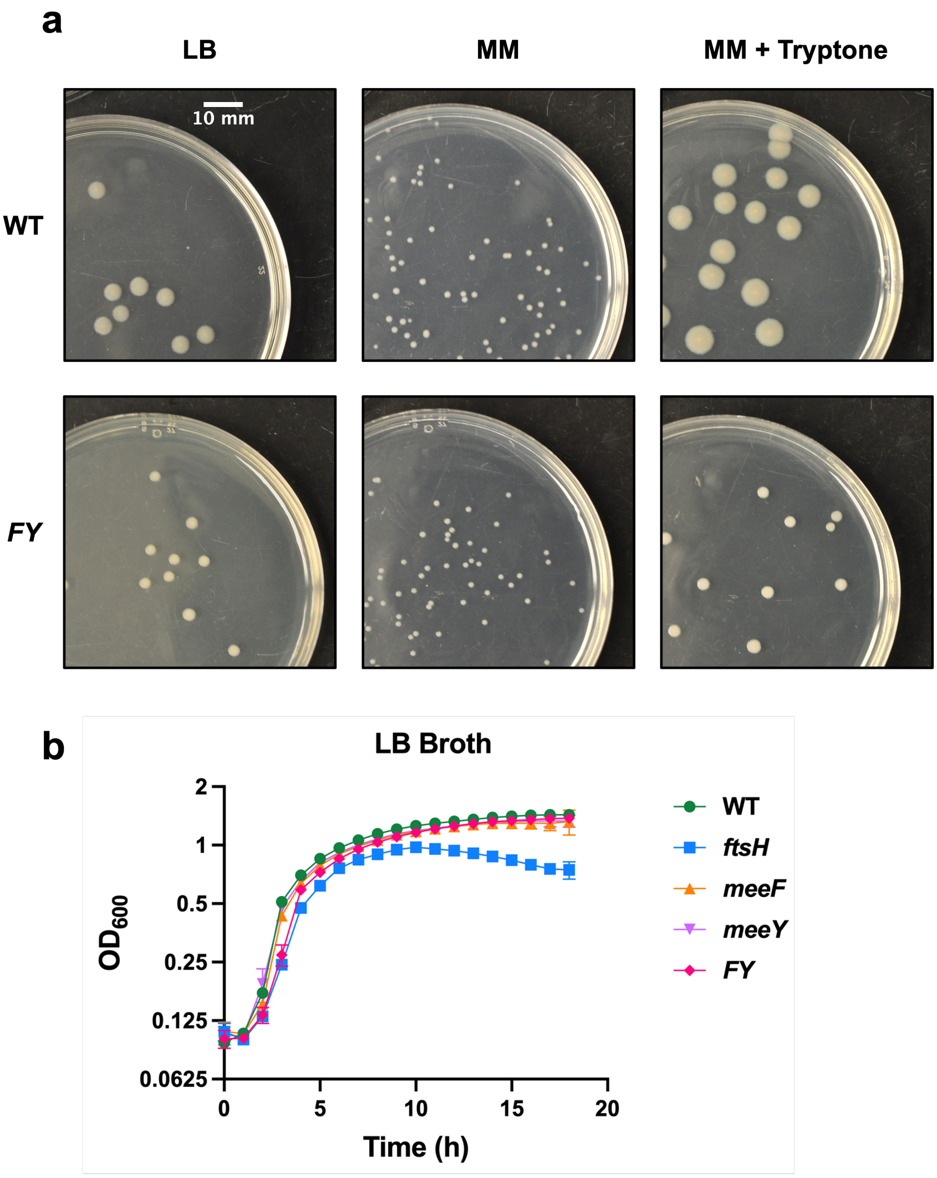

Supplementary Figure 1. Comparative growth of WT and FY mutants. (**a**) Growth of WT and FY mutants on LB and defined (MM) agar plates. Representative pictures of colony size on different medium from three independent experiments are shown. Scale bar (10 mm) is indicated and applies across all images. (**b**) Aerobic growth in liquid LB medium with shaking at 37˚C in a 96-well plate reader (Bio-Tek). Representative growth curves of different strains (WT, *ftsH, meeF, meeY, FY*) in liquid LB broth from three independent experiments are shown. Changes in OD_{600} were measured every hour. Data are presented as mean ± standard deviation with the sample number *n* = 3. Source data are provided as a Source Data file.

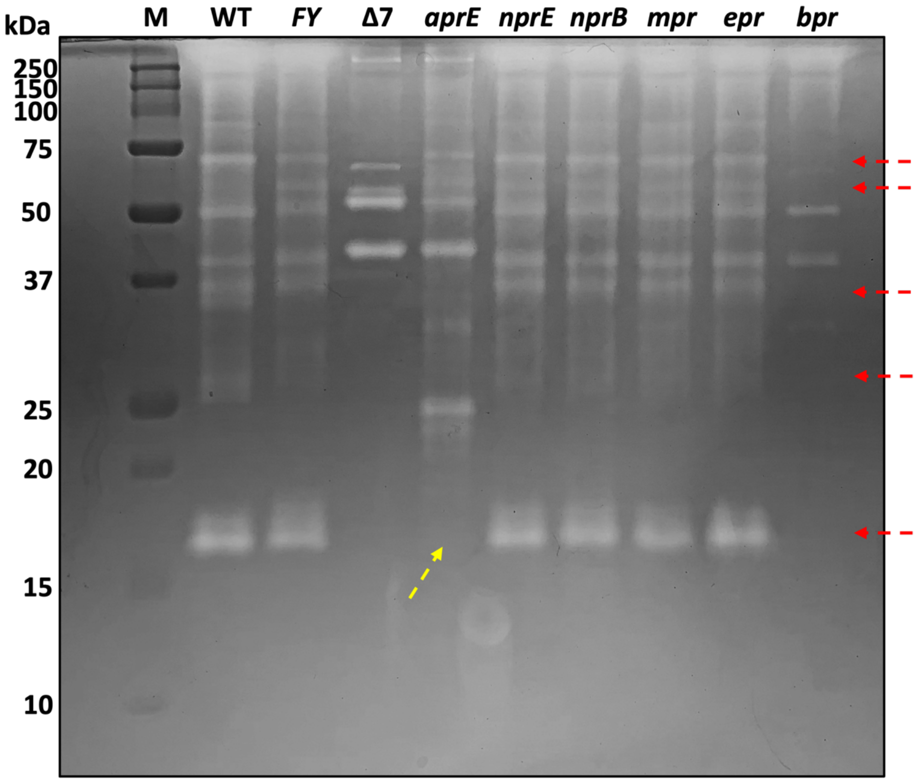

Supplementary Figure 2. Extracellular protease activities in the supernatants of mutant strains. Extracellular protease activities in the supernatants were detected by gelatin zymography. Supernatants were from overnight cultures with equal cell density. Higher protease activities correspond to clearer bands on the gel. Several bands lacking in the *bpr* null mutant were previously assigned as processed products of the large Bpr protease (red arrows)[9]. The 17 kDa processed Bpr product is also missing in the *aprE* mutant (yellow arrow), suggesting that the AprE protease is involved in Bpr processing, as suggested[9]. The presence of the 17 kDa band in the FY mutant suggests that both Bpr and AprE are still secreted in this strain, although overall protease activity is reduced. The image is representative of three independent experiments. Source data are provided as a Source Data file.

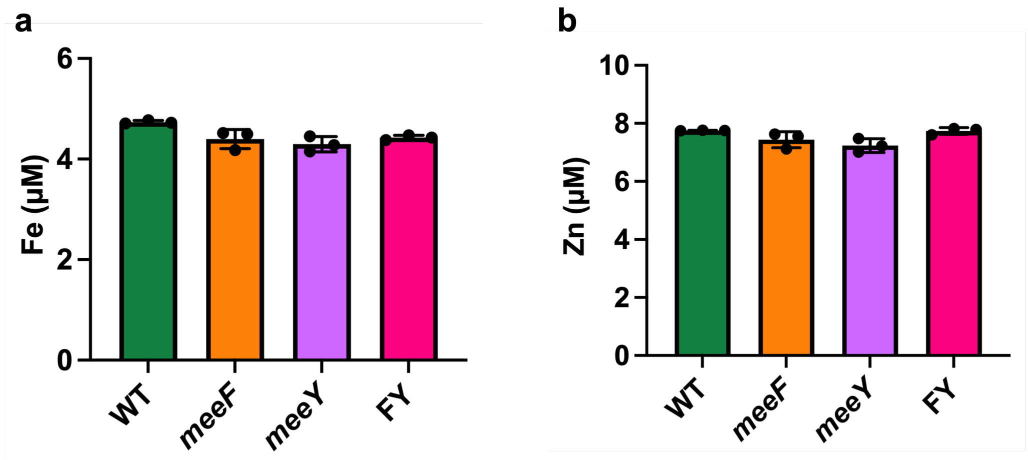

C. Table. Metal levels (µM) in LB or spent supernatants detected by ICP-MS

	**LB broth**	**WT**	***meeF***	***meeY***	***FY***
**Mn**	0.126±0.009	0.022±0.002	0.017±0.004	0.021±0.003	0.003±0.001
**Fe**	5.05±0.62	4.73±0.04	4.40±0.19	4.30±0.15	4.43±0.05
**Zn**	8.20±0.87	7.75±0.01	7.43±0.27	7.24±0.24	7.74±0.12

Supplementary Figure 3. Metal ion levels (µM) measured in cell supernatant fractions. Overnight grown cells were used to collect supernatants and Fe (**a**) and Zn (**b**) levels were detected using ICP-MS analysis. Data is from three independent experiments and presented as mean ± standard deviation. In contrast with Mn levels (Fig. 2b), there was little change in residual Fe and Zn in the supernatant fractions. (**c**) Summary Table: Samples were collected and analyzed as in Fig. 2b, Supplementary Fig. 3a, 3b. Samples were from three independent experiments and data are presented as mean ± standard deviation. Source data are provided as a Source Data file.

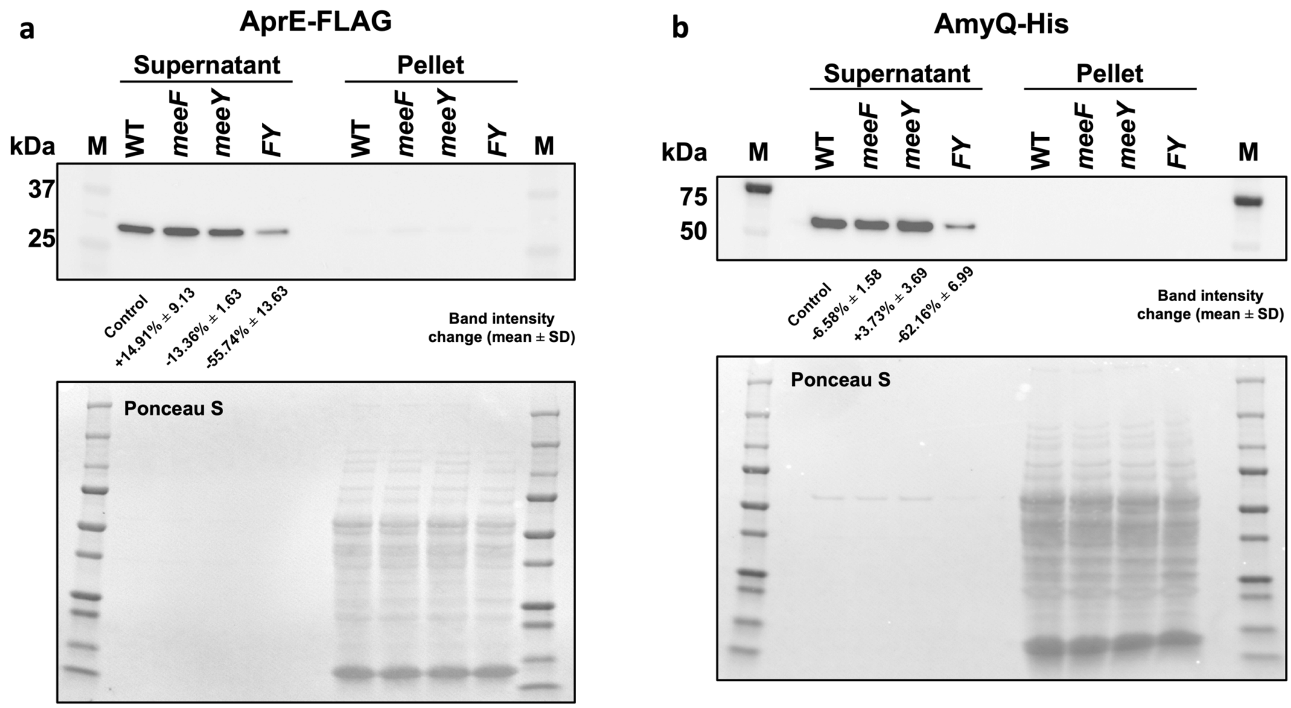

Supplementary Figure 4. Effect of *meeF*, *meeY* and FY mutations on AprE and AmyQ secretion. (**a**) The level of AprE-FLAG was measured using immunoblotting. (**b**) Defective secretion of heterologous (AmyQ-His) protein is depicted for FY. In (**a**) and (**b**), band intensity change was calculated as “change = (sample - control) / control * 100%”. Sample collection was described in Fig. 3. The protein membranes were stained by Ponceau S after protein transfer. Ponceau S-stained images (lower panels) serve as loading controls and were imaged using a GelDoc gel imaging system. Images are representative of three independent experiments and band intensities are presented as mean ± standard deviation, n = 3. Source data are provided as a Source Data file.

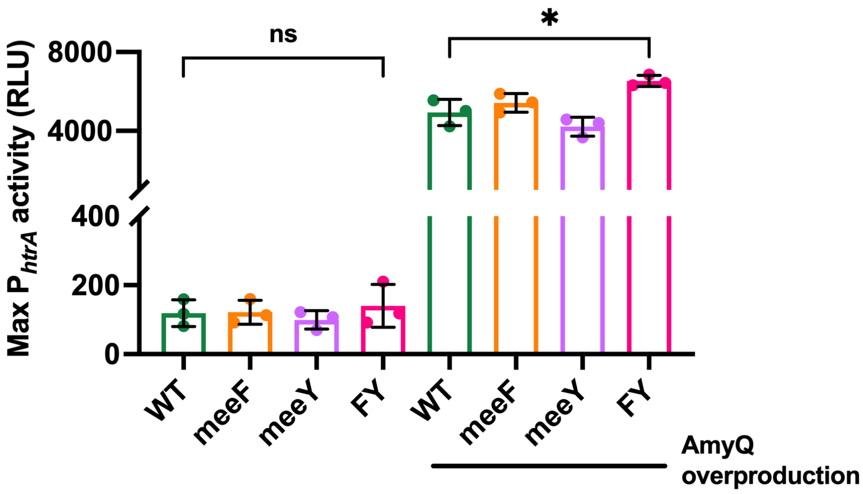

Supplementary Figure 5. Maximum P_{htrA}-lux promoter activity in stains with or without AmyQ overexpression. Expression of the CssRS secretion stress response was monitored using a $P_{htrA\text{-}lux}$ transcriptional reporter. Cells were grown in LB medium at 37˚C and the peak expression noted. AmyQ was overexpressed in the strains from pKTH10[7]. Data are presented as mean ± standard deviation, $n = 3$. ns, no significant difference, $p = 0.6498$; *, $p = 0.0374$, P value was calculated using Welch's t test, two-tailed. Source data are provided as a Source Data file.

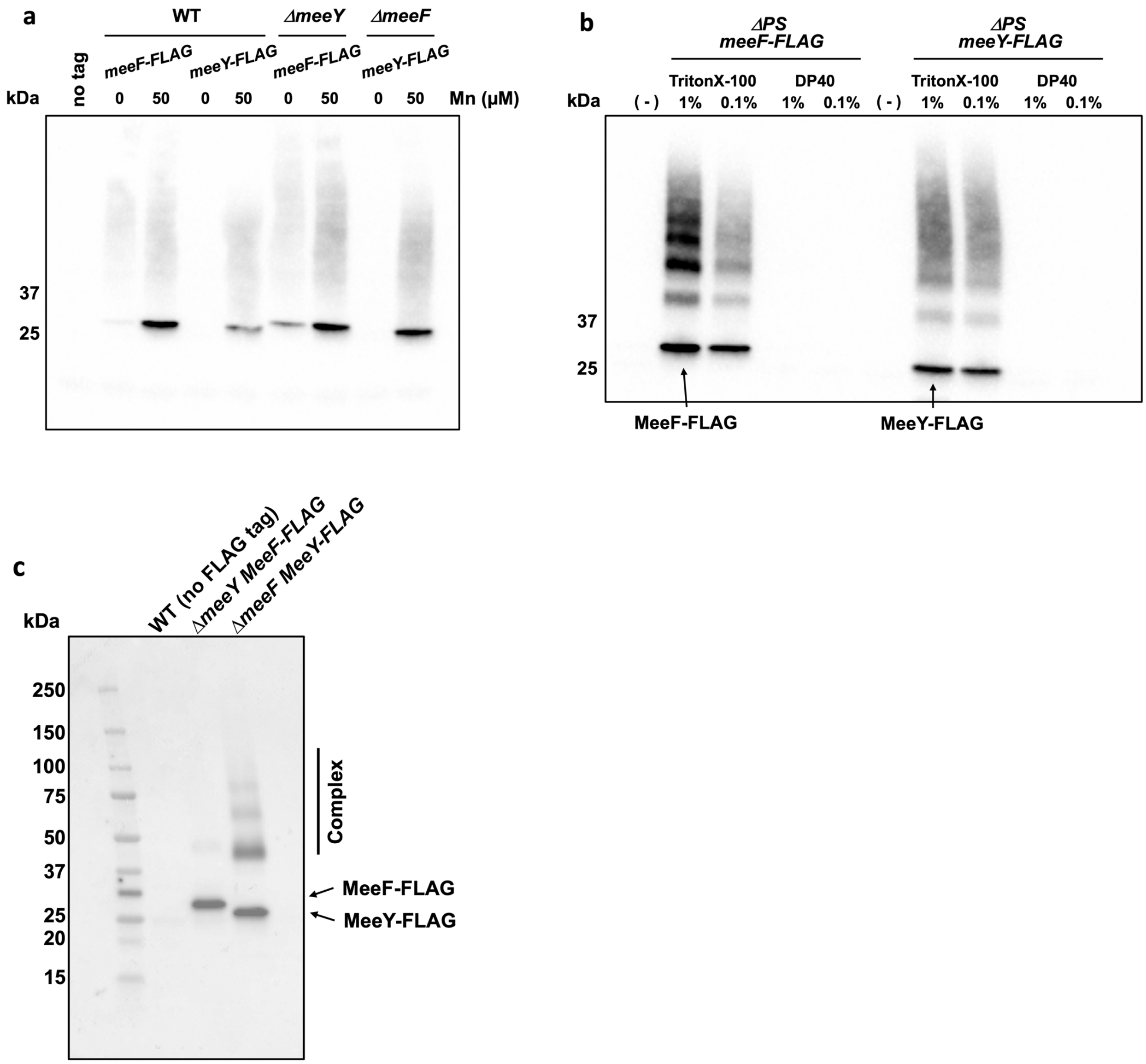

Supplementary Figure 6. MeeF-FLAG and MeeY-FLAG levels in different mutants. (**a**) MeeF-FLAG or MeeY-FLAG levels in different stain backgrounds as measured using immunoblotting. Cells were collected from overnight cultures with or without 50 μM Mn. (**b**) Representative immunoblots of MeeF-FLAG and MeeY-FLAG co-IP samples in an efflux-deficient *mneP mneS* (Δ*PS*) background. Different detergents were used for cell lysis (1% or 0.1% Triton X-100, and 1% or 0.1% DP40). Proteins were eluted from magnetic beads by heating at 95˚C for 10 min. (-) WT is the control without FLAG tag. (**c**) Co-Immunoprecipitation

samples from MeeF-FLAG and MeeY-FLAG strains were visualized by immunoblotting following pull down of cell lysates (prepared with 1% Triton X-100) using anti-FLAG-magnetic beads and subsequent elution. The lower mobility bands represent complexes stable during electrophoresis. The WT strain without a FLAG tag was used as a negative control to identify false positive protein interactions. Proteins identified in the immunoprecipitate (and not present in the corresponding fraction from the untagged control strain) are considered interacting protein partners for MeeF-FLAG or MeeY-FLAG. Source data are provided as a Source Data file.

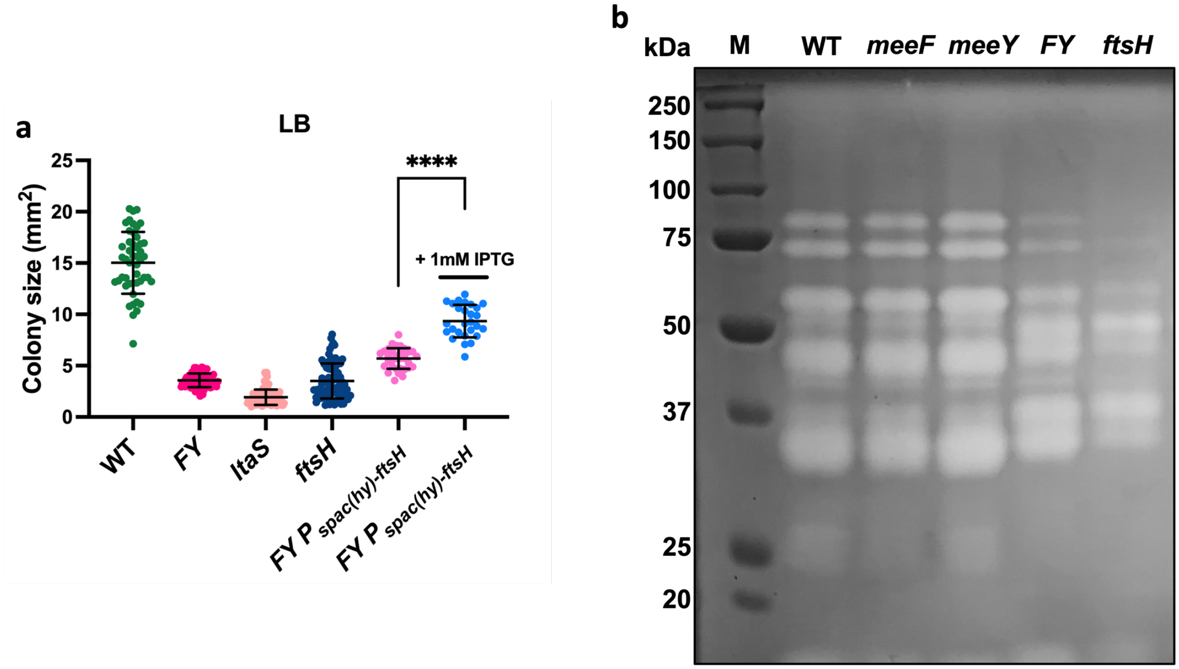

Supplementary Figure 7. Comparative phenotypes of mutations affecting protein secretion. (**a**) Colony size (mean ± standard deviation) of the indicated strains on LB medium. Isolated colonies from two independent experiments were measured. Sample size: WT, *n* = 48; FY, 76; *ltaS,* 86; *ftsH*, 82; FY $P_{spac(hy)\text{-}ftsH}$, 34; FY $P_{spac(hy)\text{-}ftsH}$ with 1 mM IPTG, 27. Growth of the FY mutant strain with $P_{spac(hy)}$-*ftsH* was significantly improved upon addition of 1 mM IPTG (****, $p < 0.0001$). *P* value was calculated using Welch's t test, two-tailed. (**b**) Protease secretion as monitored by zymography. Supernatants were collected from overnight cultures with equal cell density. Note that the FY mutant and the *ftsH* mutant both have reduced protease activities relative to the WT strain. The image is representative of three independent experiments. Source data are provided as a Source Data file.

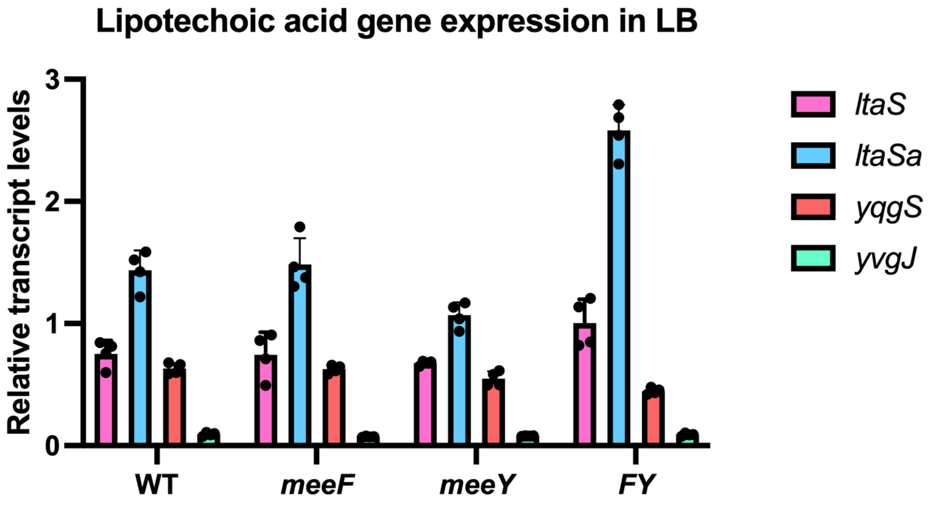

Supplementary Figure 8. Relative transcription level of LTA synthase genes in different strain backgrounds. Transcript levels of genes encoding LTA synthases (*ltaS*, *ltaSa*, *yqgS)* and LTA primase *(yvgJ)* were monitored in different stains (WT, *meeF*, *meeY* and FY) using q-RT-PCR and normalized to the *gyrA* transcript. Values shown are mean ± standard deviation from four independent experiments. FY mutants showed similar *ltaS* expression and higher *ltaSa* transcription levels compared to WT, which is consistent with the hypothesis that FY mutant has normal expression of *ltaS* but decreased LtaS activity. Source data are provided as a Source Data file.

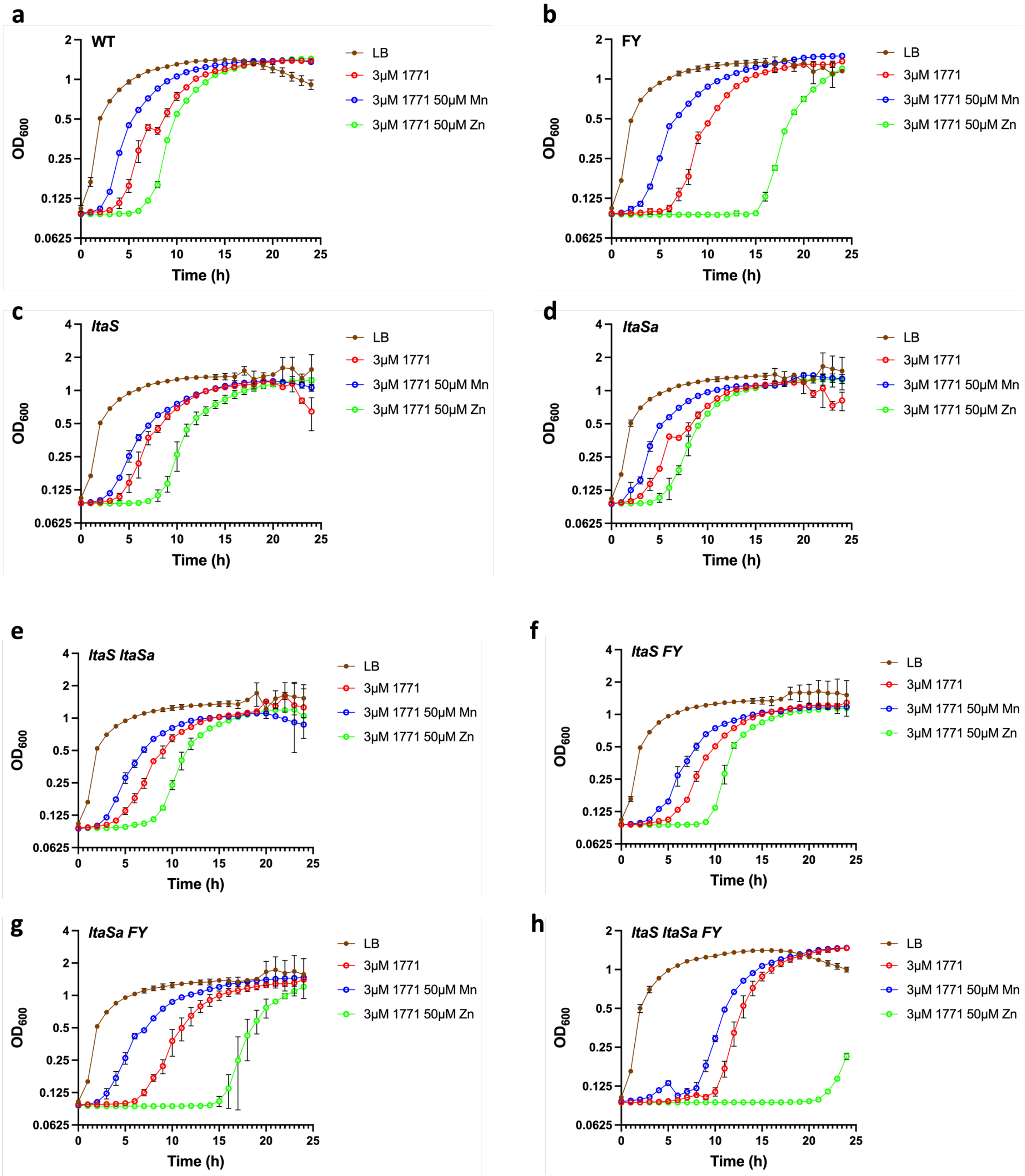

Supplementary Figure 9. Effects of metal ions of sensitivity to the LtaS inhibitor 1771. (**a**)-(**h**), Aerobic growth of different strains in LB broth with or without 3 μM 1771, 50 μM Mn or 50 μM Zn. Data are representative of three independent cultures and presented as mean ± standard deviation with sample size *n* = 2. Source data are provided as a Source Data file.

Supplementary Table 1. Bacillus subtilis stains, primers, and plasmids used in this study.

Strain	Genotype	Construction	Reference
HB27501	*trpC2 attSPβ (WT) CU1065*	Lab strain	Lab stock
HB27502	*CU1065 ΔmeeF*	Lab strain	[1]
HB27503	*trpC2 ΔmeeY*	Lab strain	[1]
HB27504	*trpC2 ΔmeeF ΔmeeY*	Lab strain	[1]
HB27531	*trpC2 aprE::erm*	BGSC-->HB27501	This work
HB27534	*trpC2 nprB::erm*	BGSC-->HB27501	This work
HB27536	*trpC2 nprE::erm*	BGSC-->HB27501	This work
HB27541	*trpC2 mpr::erm*	BGSC-->HB27501	This work
HB27560	*ΔnprE ΔaprE Δepr Δmpr ΔnprB Δvpr Δbpr*		BGSC1A1133
HB27584	*trpC2 ftsH::erm*	BGSC-->HB27501	This work
HB27637	*trpC2 bpr::erm*	BGSC-->HB27501	This work
HB27641	*trpC2 epr::erm*	BGSC-->HB27501	This work
HB27667	*trpC2 ltaS:erm*	BGSC-->HB27501	This work
HB27668	*trpC2 ΔmeeF ltaS:erm*	BGSC-->HB27502	This work
HB27669	*trpC2 ΔmeeY ltaS:erm*	BGSC-->HB27503	This work
HB27670	*trpC2 ΔFY ltaS:erm*	BGSC-->HB27504	This work
HB27680	*trpC2 sacA::*P_{htrA}*-lux-cat*	gDNA HB23650-->HB27501	[2]
HB27681	*trpC2 ΔmeeF sacA::*P_{htrA}*-lux-cat*	gDNA HB23650-->HB27502	This work
HB27682	*trpC2 ΔmeeY sacA::*P_{htrA}*-lux-cat*	gDNA HB23650-->HB27503	This work
HB27683	*trpC2 ΔFY sacA::*P_{htrA}*-lux-cat*	gDNA HB23650-->HB27504	This work
HB27684	*trpC2 amyQ-His::kan*	pKTH10-->HB27501	This work
HB27685	*trpC2 ΔmeeF amyQ-His::kan*	pKTH10 -->HB27502	This work
HB27686	*trpC2 ΔmeeY amyQ-His::kan*	pKTH10 -->HB27503	This work
HB27687	*trpC2 ΔFY amyQ-His::kan*	pKTH10[c]-->HB27504	This work
HB27721	*trpC2 aprE-FLAG::MLS*	*aprE*::pMUTIN-FLAG-->HB27501	This work
HB27722	*trpC2 ΔmeeF aprE-FLAG::MLS*	*aprE*::pMUTIN-FLAG-->HB27502	This work
HB27723	*trpC2 ΔmeeY aprE-FLAG::MLS*	*aprE*::pMUTIN-FLAG-->HB27503	This work
HB27724	*trpC2 ΔFY aprE-FLAG::MLS*	*aprE*::pMUTIN-FLAG-->HB27504	This work
HB27729	*trpC2 sacA::*P_{htrA}*-lux-cat amyQ-His::kan*	pKTH10 -->HB27680	This work
HB27730	*trpC2 ΔmeeF sacA::*P_{htrA}*-lux-cat amyQ-His::kan*	pKTH10 -->HB27681	This work
HB27731	*trpC2 ΔmeeY sacA::*P_{htrA}*-lux-cat amyQ-His::kan*	pKTH10 -->HB27682	This work
HB27732	*trpC2 ΔFY sacA::*P_{htrA}*-lux-cat amyQ-His::kan*	pKTH10 -->HB27683	This work

HB27733	*trpC2 sacA::P_{sigM}-lux-cat*	gDNA *sacA::P_{sigM}-lux*-->HB27501	[3]
HB27734	*trpC2 ΔmeeF sacA::P_{sigM}-lux-cat*	gDNA *sacA::P_{sigM}-lux*-->HB27502	This work
HB27735	*trpC2 ΔmeeY sacA::P_{sigM}-lux-cat*	gDNA *sacA::P_{sigM}-lux*-->HB27503	This work
HB27736	*trpC2 ΔFY sacA::P_{sigM}-lux-cat*	gDNA *sacA::P_{sigM}-lux*-->HB27504	This work
HB27739	*trpC2 ltaS::erm sacA::P_{sigM}-lux-cat*	gDNA *sacA::P_{sigM}-lux*-->HB27667	This work
HB27740	*trpC2 ltaS::erm ΔmeeF sacA::P_{sigM}-lux-cat*	gDNA *sacA::P_{sigM}-lux*-->HB27668	This work
HB27741	*trpC2 ltaS::erm ΔmeeY sacA::P_{sigM}-lux-cat*	gDNA *sacA::P_{sigM}-lux*-->HB27669	This work
HB27742	*trpC2 ltaS::erm ΔFY sacA::P_{sigM}-lux-cat*	gDNA *sacA::P_{sigM}-lux*-->HB27670	This work
HB27744	*trpC2 ltaSa::spec*	gDNA *ltaSa::spec*-->HB27501	[4]
HB27745	*trpC2 ΔmeeF ltaSa::spec*	gDNA HB27744-->HB27502	This work
HB27746	*trpC2 ΔmeeY ltaSa::spec*	gDNA HB27744-->HB27503	This work
HB27747	*trpC2 ΔFY ltaSa::spec*	gDNA HB27744-->HB27504	This work
HB27750	*trpC2 ltaS::erm ltaSa::spec*	gDNA HB27744-->HB27667	This work
HB27751	*trpC2 ΔmeeF ltaS::erm ltaSa::spec*	gDNA HB27744-->HB27668	This work
HB27752	*trpC2 ΔmeeY ltaS::erm ltaSa::spec*	gDNA HB27744-->HB27669	This work
HB27753	*trpC2 ΔFY ltaS::erm ltaSa::spec*	gDNA HB27744-->HB27670	This work
HB27770	*trpC2 amyE::$P_{spac(hy)}$-meeF-cat*	pPL82-*meeF*-->HB27501	This work
HB27772	*trpC2 amyE::$P_{spac(hy)}$-lmo0991-cat*	pPL82-lmo0991-->HB27501	This work
HB27773	*trpC2 amyE::$P_{spac(hy)}$-lmo0992-cat*	pPL82-lmo0992-->HB27501	This work
HB27774	*trpC2 amyE::$P_{spac(hy)}$-BanTerC-cat*	pPL82-lmoBanTerC-->HB27501	This work
HB27783	*trpC2 ΔFY amyE::$P_{spac(hy)}$-meeF-cat*	pPL82-*meeF*-->HB27504	This work
HB27784	*trpC2 ΔFY amyE::$P_{spac(hy)}$-lmo0991-cat*	pPL82-lmo0991-->HB27504	This work
HB27785	*trpC2 ΔFY amyE::$P_{spac(hy)}$-lmo0992-cat*	pPL82-lmo0992-->HB27504	This work
HB27787	*trpC2 ΔFY amyE::$P_{spac(hy)}$-BanTerC-cat*	pPL82-lmoBanTerC-->HB27504	This work
HB27788	*trpC2 ltaSa::spec sacA::P_{sigM}-lux-cat*	gDNA *sacA::P_{sigM}-lux*-->HB27744	This work
HB27789	*trpC2 ΔmeeF ltaSa::spec sacA::P_{sigM}-lux-cat*	gDNA *sacA::P_{sigM}-lux*-->HB27745	This work
HB27790	*trpC2 ΔmeeY ltaSa::spec sacA::P_{sigM}-lux-cat*	gDNA *sacA::P_{sigM}-lux*-->HB27746	This work
HB27791	*trpC2 ΔFY ltaSa::spec sacA::P_{sigM}-lux-cat*	gDNA *sacA::P_{sigM}-lux*-->HB27747	This work
HB27792	*trpC2 ltaS::erm ltaSa::spec sacA::P_{sigM}-lux-cat*	gDNA *sacA::P_{sigM}-lux*-->HB27750	This work
HB27832	*amyE::$P_{spac(hy)}$-meeY-cat*	pPL82-*meeY*-->HB27501	This work
HB27834	*trpC2 ΔmeeY amyE::$P_{spac(hy)}$-meeY-cat*	pPL82-*meeY*-->HB27503	This work
HB27836	*trpC2 ΔFY amyE::$P_{spac(hy)}$-meeY-cat*	pPL82-*meeY*-->HB27504	This work
HBAS924	*trpC2 meeF-FLAG::MLS*	pMUTIN-FLAG	This work
HBAS943	*trpC2 meeY-FLAG::MLS*	pMUTIN-FLAG	This work

HBAS927	*trpC2 ΔmeeY meeF-FLAG::MLS*	pMUTIN-FLAG	This work
HBAS949	*trpC2 ΔmeeF meeY-FLAG::MLS*	pMUTIN-FLAG	This work
HBAS930	*trpC2 ΔmneP ΔmneS meeF-FLAG::MLS*	pMUTIN-FLAG	This work
HBAS946	*trpC2 ΔmneP ΔmneS meeY-FLAG::MLS*	pMUTIN-FLAG	This work

**Primer name**	**Sequence**	**Purpose**
meeFcheckF	GTGTCATCCATACGGGTGACAA	Deletion check
meeFcheckR	GATTTGAGTGCTTCGCAATCAGCT	
meeYcheckF	GGAGGAAGGCCGTTTCTTGA	Deletion check
meeYcheckR	CGGATGAAACCGTCTTTGCG	
aprEcheckF	CGAGTCTCTACGGAAATAGCGAG	Deletion check
aprEcheckR	CTTGTGAAGATTTTCAGAGGCAGC	
nprBcheckF	GCAGCTCATACCTCCGCTTATAC	Deletion check
nprBcheckR	GATTGGAGCAATCAAACCGTCG	
nprEcheckF	CTCTTGAATGAACCACCACATGAC	Deletion check
nprEcheckR	ATAGCCACGTGACCTGTAGC	
mprcheckF	GCTTGACACTAAAGGAGGGAGATG	Deletion check
mprcheckR	GAAGAATGCGTCTGCGAACTG	
eprcheckF	CACCCGAGTGAATGTGCTCAT	Deletion check
eprcheckR	CATCATTGGGTCTTGCCTGC	
bprcheckF	CAGCGATGTTCTGACAAACCATTC	Deletion check
bprcheckR	TGCCGTGAGCAAAAGCAAAAG	
ftsHcheckF	CTGAGCGCTATCGCAATCTG	Deletion check
ftsHcheckR	TACACGATCAGCGGCTCA	
ltaScheckF	GCGAAACGTTGATTCGACGG	Deletion check
ltaScheckR	GCTGAGGAATTGAGGGCTG	
ftsH700F	ATTGTGACGGACGCAAGCGGTGAAATTATC	PCR insertion
ftsH700R	TCCTGTCGCAATGACCTTTGGTTCCTGT	
aprEFLAGHindIIIF	ATATAAGCTTTCTCACGGCACACATGTAGCCG	Cloning in pMUTIN-FLAG
aprEFLAGKpnIR	ATATGGTACCTTGTGCAGCTGCTTGTACGTTG	
meeFXbalF	CAGTTCTAGAAAAGGAGGAAGGATCATTGGACTTTTTACATCATAT TTTGTCTACG	Cloning in pPL82
meeFBglIIR	CGTTAGATCTTTATTCTTCTTTTGAAGCGGCTGTTT	
meeYXabIF	ATATTCTAGACAAATCACTGCGCTGCGCATATTATTG	Cloning in pPL82
meeYBglIIR	CGTTAGATCTTTACGCCCGTTCACGGGTGCTG	
lmo0991XbalF	CAGTTCTAGAAAAGGAGGAAGGATCAATGGATACAGCAATGATTTT AGAGTAC	Cloning in pPL82
lmo0991BglIIR	CGTTAGATCTTTATTTAGTTGTTTCTGTTTTTTTCTTAC	
lmo0992XbalF	CAGTTCTAGAAAAGGAGGAAGGATCAATGGATGTTTCTATTTGGG GCGAATATG	Cloning in pPL82[5]

lmo0992BglIIR	CGTTAGATCTTCAAACTCTTTTGGTTTTCTTCTC	
BanTerCXbalF	CAGTTCTAGAAAAGGAGGAAGGATCAATGAGTATTTTGCAAGGAATCCTTGATAC	Cloning in pPL82
BanTerCBglIIR	CGTTAGATCTTTATTTATGGTTATTTTTAGTAGCTGCAAC	
ftsHXbalF	CAGTTCTAGAAAAGGAGGAAGGATCAATGAATCGGGTCTTCCGTAATACCATTTTT	Cloning in pPL82
ftsHBglIIR	CGTTAGATCTTTACTCTTTCGTATCGTCTTTCTTTTCTTCTGTT	
meeF-FLAG-HindIII-F	ATATAAGCTTTGGTGGATCAAGGTGCTTGGCGCGCTTTACCTGGCTTG	Cloning in pMUTIN-FLAG
meeF-FLAG-KpnI-R	ATATGGTACCTTCTTCTTTTGAAGCGGCTGTTTGTTCGCGCAC	Cloning in pMUTIN-FLAG
meeY-FLAG-KpnI-F	ATATGGTACCAAGAAGACACACATAAAGAGACGAAGCAAAG	Cloning in pMUTIN-FLAG
meeY-FLAG-KpnI-R	ATATGGTACCCGCCCGTTCACGGGTGCTGTTTTTTTGTTC	Cloning in pMUTIN-FLAG
pMUTIN4-FLAG check-F	ACATCCAGAACAACCTCTGCTAAAATTC	Cloning check; pMUTIN-FLAG
ltaS-F-qPCR	TTCAGTTTTCGTAAACAAAGCGC	qRT-PCR
ltaS-R-qPCR	ATGCCTTCCGCCTTGTACATTTC	qRT-PCR
ltaSa-F-qPCR	CAGCCATTATTATGCTGATTATCG	qRT-PCR
ltaSa-R-qPCR	GCCATGATACTGAAAATCCCGTC	qRT-PCR
yvgJ-F-qPCR	GCCAATTTGGTTCTGACTGTTATC	qRT-PCR
yvgJ-R-qPCR	CTTTTACACTGCTGCCCATATCGCTC	qRT-PCR
yqgS-F-qPCR	ATGCTGATTGCCATTTTATTGATGTG	qRT-PCR
yqgS-R-qPCR	TGCCAGCAATACAAACGTGAC	qRT-PCR

**Plasmid**	**Properties**	**Reference**
pPL82	IPTG-induced overexpression construction	[6]
pKTH10	overexpression of AmyQ	[7]
pMUTIN-FLAG	tagged genes with FLAG sequence at C-terminal	[5]
pBS3C*lux*	luciferase promoter construction	[8]

Supplementary References

1. Paruthiyil, S., Pinochet-Barros, A., Huang, X. & Helmann, J.D. *Bacillus subtilis* TerC Family Proteins Help Prevent Manganese Intoxication. *J Bacteriol* **202**(2020).
2. Zhao, H., Sachla, A.J. & Helmann, J.D. Mutations of the *Bacillus subtilis* YidC1 (SpoIIIJ) insertase alleviate stress associated with σ^{M}-dependent membrane protein overproduction. *PLoS Genet* **15**, e1008263 (2019).
3. Zhao, H., Roistacher, D.M. & Helmann, J.D. Aspartate deficiency limits peptidoglycan synthesis and sensitizes cells to antibiotics targeting cell wall synthesis in *Bacillus subtilis*. *Mol Microbiol* **109**, 826-844 (2018).
4. Sachla, A.J. & Helmann, J.D. A bacterial checkpoint protein for ribosome assembly moonlights as an essential metabolite-proofreading enzyme. *Nat Commun* **10**, 1526 (2019).
5. Kaltwasser, M., Wiegert, T. & Schumann, W. Construction and Application of Epitope- and Green Fluorescent Protein-Tagging Integration Vectors for *Bacillus subtilis*. *Applied and Environmental Microbiology* **68**, 2624-2628 (2002).
6. Quisel, J.D., Burkholder, W.F. & Grossman, A.D. In vivo effects of sporulation kinases on mutant Spo0A proteins in *Bacillus subtilis*. *Journal of bacteriology* **183**, 6573-6578 (2001).
7. Palva, I. Molecular cloning of alpha-amylase gene from *Bacillus amyloliquefaciens* and its expression in *B. subtilis. Gene* **19**, 81-7 (1982).
8. Radeck, J. et al. The Bacillus BioBrick Box: generation and evaluation of essential genetic building blocks for standardized work with *Bacillus subtilis*. *J Biol Eng* **7**, 29 (2013).
9. Kodama, T., Endo, K., Ara, K., Ozaki, K. & Sekiguchi, J. Zymography of extracellular proteases in *Bacillus subtilis*. *Int. J. Biosci. Biotechnol.* **1**, 60-66 (2013).
